# Metabolic network alterations as a supportive biomarker in dementia with Lewy bodies with preserved dopamine transmission

**DOI:** 10.1007/s00259-023-06493-w

**Published:** 2023-11-16

**Authors:** Anna Stockbauer, Leonie Beyer, Maria Huber, Annika Kreuzer, Carla Palleis, Sabrina Katzdobler, Boris-Stephan Rauchmann, Silvia Morbelli, Andrea Chincarini, Rose Bruffaerts, Rik Vandenberghe, Milica G. Kramberger, Maja Trost, Valentina Garibotto, Nicolas Nicastro, Aurélien Lathuilière, Afina W. Lemstra, Bart N. M. van Berckel, Andrea Pilotto, Alessandro Padovani, Miguel A. Ochoa-Figueroa, Anette Davidsson, Valle Camacho, Enrico Peira, Matteo Bauckneht, Matteo Pardini, Gianmario Sambuceti, Dag Aarsland, Flavio Nobili, Mattes Gross, Jonathan Vöglein, Robert Perneczky, Oliver Pogarell, Katharina Buerger, Nicolai Franzmeier, Adrian Danek, Johannes Levin, Günter U. Höglinger, Peter Bartenstein, Paul Cumming, Axel Rominger, Matthias Brendel

**Affiliations:** 1 Department of Nuclear Medicine, University Hospital, LMU Munich, Munich, Germany; 2 Department of Neurology, University Hospital, LMU Munich, Munich, Germany; 3 German Center for Neurodegenerative Diseases (DZNE), Munich, Germany; 4 Munich Cluster for Systems Neurology (SyNergy), Munich, Germany; 5 Department of Psychiatry and Psychotherapy, University Hospital, LMU Munich, Munich, Germany; 6 Department of Neuroradiology, University Hospital of Munich, LMU Munich, Munich, Germany; 7 Nuclear Medicine Uni, IRCCS Ospedale Policlinico San Martino, Genoa, Italy; 8 Department of Health Sciences, University of Genoa, Genoa, Italy; 9 National Institute of Nuclear Physics (INFN), Genoa Section, Genoa, Italy; 10 Laboratory for Cognitive Neurology, Department of Neurosciences, KU Leuven, Louvain, Belgium; 11 Neurology Department, University Hospitals Leuven, Louvain, Belgium; 12 Biomedical Research Institute, Hasselt University, Hasselt, Belgium; 13 Experimental Neurobiology Unit, Department of Biomedical Sciences, University of Antwerp, Antwerp, Belgium; 14 Department of Neurology and Department for Nuclear Medicine, University Medical Centre, Ljubljana, Slovenia; 15 Faculty of Medicine, University of Ljubljana, Ljubljana, Slovenia; 16 Division of Nuclear Medicine and Molecular Imaging, Geneva University Hospitals and NIMTLab, Geneva University, Geneva, Switzerland; 17 Department of Clinical Neurosciences, Geneva University Hospitals, Geneva, Switzerland; 18 LANVIE (Laboratoire de Neuroimagerie du Vieillissement), Department of Psychiatry, Geneva University Hospitals, Geneva, Switzerland; 19 Alzheimer Center Amsterdam, Department of Neurology, Amsterdam Neuroscience, Vrije Universiteit Amsterdam, Amsterdam UMC, Amsterdam, The Netherlands; 20 Department of Radiology & Nuclear Medicine, Amsterdam Neuroscience, Vrije Universiteit Amsterdam, Amsterdam UMC, Amsterdam, The Netherlands; 21 Neurology Unit, Department of Clinical and Experimental Sciences, University of Brescia, Brescia, Italy; 22 Parkinson’s Disease Rehabilitation Centre, FERB ONLUS - S. Isidoro Hospital, Trescore Balneario, BG Italy; 23 Department of Clinical Physiology in Linköping, Department of Health, Medicine and Caring Sciences, Linköping University, Linköping, Sweden; 24 Department of Diagnostic Radiology, Linköping University Hospital, Linköping, Sweden; 25 Center for Medical Image Science and Visualization (CMIV), Linköping University, Linköping, Sweden; 26 Servicio de Medicina Nuclear, Hospital de la Santa Creu i Sant Pau, Universitat Autònoma de Barcelona, Barcelona, Spain; 27 Department of Neuroscience (DINOGMI), University of Genoa, Genoa, Italy; 28 Clinical Neurology, IRCCS Ospedale Policlinico San Martino, Genoa, Italy; 29 Centre of Age-Related Medicine (SESAM), Stavanger University Hospital, Stavanger, Norway; 30 Department of Old Age Psychiatry, Institute of Psychiatry, Psychology, and Neuroscience, King’s College, London, UK; 31 Sheffield Institute for Translational Neuroscience (SITraN), University of Sheffield, Sheffield, S10 2HQ UK; 32 Ageing Epidemiology (AGE) Research Unit, School of Public Health, Imperial College, London, UK; 33 Institut for Stroke and Dementia Research, University of Munich, Munich, Germany; 34 Department of Nuclear Medicine, University of Bern, Inselspital Bern, Bern, Switzerland; 35 School of Psychology and Counselling and IHBI, Queensland University of Technology, Brisbane, Australia

**Keywords:** Dementia with Lewy bodies, FDG-PET, Metabolic connectivity, DaT-Scan

## Abstract

**Purpose:**

Metabolic network analysis of FDG-PET utilizes an index of inter-regional correlation of resting state glucose metabolism and has been proven to provide complementary information regarding the disease process in parkinsonian syndromes. The goals of this study were (i) to evaluate pattern similarities of glucose metabolism and network connectivity in dementia with Lewy bodies (DLB) subjects with subthreshold dopaminergic loss compared to advanced disease stages and to (ii) investigate metabolic network alterations of FDG-PET for discrimination of patients with early DLB from other neurodegenerative disorders (Alzheimer’s disease, Parkinson’s disease, multiple system atrophy) at individual patient level via principal component analysis (PCA).

**Methods:**

FDG-PETs of subjects with probable or possible DLB (*n* = 22) without significant dopamine deficiency (*z*-score < 2 in putamen binding loss on DaT-SPECT compared to healthy controls (HC)) were scaled by global-mean, prior to volume-of-interest-based analyses of relative glucose metabolism. Single region metabolic changes and network connectivity changes were compared against HC (*n* = 23) and against DLB subjects with significant dopamine deficiency (*n* = 86). PCA was applied to test discrimination of patients with DLB from disease controls (*n* = 101) at individual patient level.

**Results:**

Similar patterns of hypo- (parietal- and occipital cortex) and hypermetabolism (basal ganglia, limbic system, motor cortices) were observed in DLB patients with and without significant dopamine deficiency when compared to HC. Metabolic connectivity alterations correlated between DLB patients with and without significant dopamine deficiency (*R*^2^ = 0.597, *p* < 0.01). A PCA trained by DLB patients with dopamine deficiency and HC discriminated DLB patients without significant dopaminergic loss from other neurodegenerative parkinsonian disorders at individual patient level (area-under-the-curve (AUC): 0.912).

**Conclusion:**

Disease-specific patterns of altered glucose metabolism and altered metabolic networks are present in DLB subjects without significant dopaminergic loss. Metabolic network alterations in FDG-PET can act as a supporting biomarker in the subgroup of DLB patients without significant dopaminergic loss at symptoms onset.

**Supplementary Information:**

The online version contains supplementary material available at 10.1007/s00259-023-06493-w.

## Introduction

In dementia with Lewy bodies (DLB), cognitive impairment and fluctuating cognition [1, 2] can occur together with a varying subset of the other characterizing core symptoms, i.e. parkinsonism, visual hallucinations and rapid eye movement (REM) sleep behaviour disorder [3, 4]. The overlap of clinical symptoms with prodromal stages of other α-synuclein-related syndromes such as Parkinson’s diseases (PD) [5] or multiple system atrophy (MSA) [6], as well as Alzheimer’s disease (AD) [7], complicates clinical diagnosis and calls for additional biomarkers [8].

In the recently published research criteria for the diagnosis of prodromal DLB [8], reduced dopamine transporter (DAT) uptake in basal ganglia represents one of the proposed biomarkers together with polysomnographic confirmation of REM sleep without atonia and reduced meta-iodobenzylguanidine uptake on myocardial scintigraphy. In patients with MCI and clinically diagnosed probable or possible DLB, reduced dopamine availability showed a high specificity of 89% in distinguishing prodromal DLB from prodromal AD, but only a sensitivity of 54%. This indicated that many clinical suspected DLB patients do not show reduced dopamine availability at an early disease stage and even at later points in the disease process [9, 10]. Normal DaT-SPECT findings could therefore cause diagnostic uncertainty and even lead to misdiagnosis [11]. Recent research has even evaluated the hypothesis whether patients without pathological DaT-SPECT should be classified as an entirely different endophenotype of DLB, making correct DLB diagnosis even more challenging in clinical practise [12].

In PD and atypical parkinsonian syndromes such as MSA, progressive supranuclear palsy and corticobasal syndrome, metabolic network analysis in 2-Fluor-2-desoxy-D-glucose positron-emission-tomography (FDG-PET) has been proven to provide complementary information to dopamine deficiency underlying the disease process [13, 14]. In this regard, metabolic network connectivity provides an index of inter-regional correlation of resting state glucose metabolism [15]. In patients with DLB, only few investigations focused on FDG-PET in the context of lacking dopaminergic deficit at symptom onset [16]. Reduced occipital glucose metabolism together with a relative preservation of posterior cingulate metabolism (known as the cingulate island sign) has been described for DLB [17]. Decreased dopamine availability has been shown to correlate with relative glucose hypometabolism in occipital and parietal regions, relative glucose hypermetabolism in basal ganglia and limbic system and impaired metabolic connectivity within those disease-related brain regions [18]. Connectivity alterations have been detected in prodromal phases of REM-sleep behavioural disorder (iRBD) before DLB diagnosis becomes evident [19]. These network-level alterations were regionally associated with the core clinical criteria for DLB [20]. Recently, a newly identified DLB-related pattern (DLBRP) of metabolic activity has proven to distinguish DLB patients from healthy controls (HC) [21]. Thus, metabolic alterations in DLB with preserved dopamine transmission could potentially be used as an additional biomarker and distinguishing criterion for supporting early clinical diagnosis of DLB.

Therefore, the goal of this study was to evaluate alterations of relative glucose metabolism and metabolic network connectivity in DLB patients without significant dopamine deficiency when compared to DLB patients with significant dopamine deficiency. We explored the discriminatory power of FDG-PET through region-based and network-based analyses comparing DLB patients with and without significant dopamine deficiency against healthy controls. For transfer into a clinical setting, we further challenged FDG-PET metabolic network alterations at the individual patient level. We used DLB patients with dopamine deficiency and healthy controls to train a single subject pattern expression score based on a principal component analysis (PCA) which was subsequently tested for discrimination of DLB patients without significant dopaminergic loss from patients with other neurodegenerative diseases (PD, MSA, AD).

## Material and methods

### E-DLB consortium: study design and patient selection

The framework of the European dementia with Lewy bodies (E-DLB) consortium with conception, design and patient selection has been described previously [22]. Within the imaging arm of this study, all patients with available brain FDG-PET scan and additional DaT-SPECT images were included [18] together with additional datasets acquired at LMU Munich between 02/2018 and 10/2019 resulting in a total of 108 patients with DLB. DLB diagnosis was based on the established criteria [4]. In order to minimalize the risk of misdiagnosis, we only included imaging data of patients with initially both probable and possible DLB diagnosis and with cognitive impairment both in the prodromal and the dementia stage who received confirmation of DLB diagnosis based on clinical follow-up in experienced neurological centres. FDG-PET images of 23 HCs imaged in Munich (*n* = 9) and Genova (*n* = 14) and DaT-SPECTs of 37 historical similarly aged HC served as controls [23].

For validation of the PCA expression score at the individual patient level, a cohort of patients with a confirmed diagnosis of either AD, PD or MSA were included. FDG-PET scans of this validation cohort have all been acquired previously at LMU Munich in the same time period [24]. Included subjects were 19 patients with MSA (mean age 63.8), 33 patients with PD (mean age 77.7) and 49 patients with AD (mean age 69.7). All patients with AD had a positive amyloid-PET scan and at least minor perfusion alterations (A+/N+). Diagnoses were made by a team of experienced clinicians based on current diagnosis criteria and confirmed through imaging and laboratory parameters as well as clinical follow-up [25–28]. Details on healthy controls as well as patients with AD, PD and MSA are provided in Supplemental Table 1.

Patients all gave informed written consent for the diagnostic procedures including radiopharmaceutical applications. Local institutional ethics committees approved the retrospective analyses and transfer of imaging data separately for all centres.

### Image acquisition and data processing

DaT-SPECT and FDG-PET images were acquired and preprocessed as described previously [18]. Details on sites and scanners are provided in Supplemental Table 2. In brief, *z*-score values against HC were calculated for the DaT-ratio in the bilateral putamen (as defined in Hermes BRASS model 5, Hermes Medical Solutions, Stockholm, Sweden) with the bilateral occipital lobe as reference region. FDG-PET images were spatially normalized and scaled to their global mean for assessment of relative regional glucose metabolism using PMOD (V3.5, PMOD technologies, Basel, Switzerland). This approach delivered robust metabolic connectivity analysis, cross-validated by intensity normalization using the cerebellum and a cluster-based approach [18]. Next, images were smoothed using a Gaussian filter (8mm^3^) and global mean scaled standardized uptake value ratios (SUVrs) were extracted for 77 predefined cortical and subcortical gray matter VOIs of the Hammers atlas [29]. The whole brain VOI was derived from merging all 77 VOIs. Eight composite regions were defined by summarizing Hammers atlas regions of frontal, parietal, temporal, occipital and insular cortex regions as well as limbic regions, basal ganglia and cerebellum within the PMOD software package.

According to the putaminal DaT-ratio *z*-scores, patients were categorized into DLB patients with significant dopamine deficiency (≥ 2 standard deviations (SD) below HC, DLB-DaT(+)) and DLB patients without significant dopamine deficiency (< 2SD below HC, DLB-DaT(−)). The average of the putaminal *z*-scores of both hemispheres served as classifier to categorize subjects in one of both groups. The control cohort was used as implemented in the Hermes software package, using age matched comparison.

### Statistical analyses

All metric values are expressed as mean ± SD. Demographics of DLB groups (DLB-DaT(+)/DLB-DaT(−)) were compared using a Student *t*-test for metric and a Fisher exact test for categorical variables.

For comparison of region-based global mean scaled FDG-PET SUVr of all 77 VOIs, one-way ANCOVAs (including age and sex as covariates) with post hoc testing (Bonferroni correction) were performed between HC, DLB-DaT(−) and DLB-DaT(+) patients and effect sizes (Cohen’s *d*) were calculated between DLB-DaT(−) and DLB-DaT(+) against HC, respectively using R (version 3.6.1, The R Foundation for Statistical Computing). The correlation of regional effect sizes was calculated between DLB-DaT(−) and DLB-DaT(+) patients.

In order to evaluate metabolic pattern similarities between DLB with and without significant dopaminergic loss, we conducted a metabolic connectivity analysis as described in our previous work by Huber et al. [18]. By calculation of inter-region correlation coefficients (ICCs, Pearson) for all pairs of global-mean scaled regional FDG-PET regional values (77 × 77 matrix), we performed a group-level metabolic connectivity analysis in each of the three subgroups (HC, Dat(+), DaT(−)), followed by Fisher’s transformation to enhance normal distribution. The difference between the Fisher transformed metabolic connectivity values of DLB-DaT(−) to HC and DLB-DaT(+) to HC of all 77 × 77 VOI pairs was calculated and the resulting indices of regional metabolic connectivity alterations were correlated with each other to analyse the similarities between the metabolic connectivity patterns of the two DLB subgroups. A supplemental voxel-based analysis between both DLB groups and controls was performed as described previously (*p* < 0.001, uncorrected for multiple comparisons) [18].

**Fig. 1:**
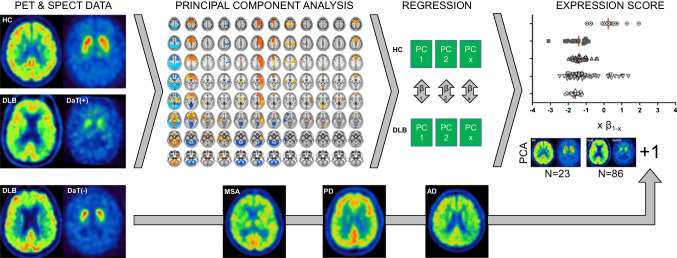
Flow-chart of principal component analysis (PCA)-based calculation of single subject DLB pattern expression. FDG-PET images of patients with DLB were stratified according to dopamine deficiency (2 SD threshold versus healthy controls). Healthy controls and DLB patients with significant dopamine deficiency were used as a PCA training cohort. The resulting principal components were subject to a linear regression with DLB status as outcome variable. Determined weighting factors from the regression (*β*) were used to calculate individual expression scores based on the single subject factor scores by adding single individuals (DBL without significant dopamine deficiency, multiple systems atrophy, Parkinson’s disease, Alzheimer’s disease) to the PCA training set (simulating a clinical scenario)

For individual subject classification, a PCA was conducted using SPSS 25 statistics (IBM Deutschland GmbH, Ehningen, Germany). Figure 1 illustrates the PCA-based calculation of single subject pattern expression. Following a previously described approach [30], the FDG-PET values of the Hammers VOI grid regions were log transformed and double centred by subtracting the mean of the data per row as well as per column of the subject in order to clear covariance and normalize the data to mean metabolic activity. We extracted components with an Eigenvalue > 1.0 and selected the Varimax rotation. Age and sex were included as covariates. The factor analysis was set to list wise exclusion of cases, suppressing small coefficients with an absolute value below 0.1 and sorting coefficients by size. Twelve principal components (PCs) resulted from the PCA for the training cohort of *n* = 23 HC and *n* = 86 DLB-DaT(+) patients. The weighting factors (regression coefficients, *β*) for each PC were determined by multiple linear regression using the DLB status as outcome variable (Supplemental Table 3). To simulate a clinical scenario, single individual subjects of the study test cohorts consisting of DLB-DaT(−), AD, PD and MSA were added into the PCA to simulate a clinical scenario. Thus, the PCA was performed 123 times each with *n* = 110 cases (*n* = 109 training plus one test case). Individual factor values of the test cases were extracted and multiplied with the aforementioned PC weighting factors, followed by subsequent summation to a single expression score per individual subject. To exclude floating of PCA expression scores by inclusion of individual test cases, the expression scores of healthy controls were extracted for each PCA run and subject to a variance analysis. PCA expression scores were then converted into *z*-scores relative to the HC group to allow for compatibility with future studies and to increase interpretability. A ROC analysis was performed for discrimination of DLB-DaT(−), AD, PD and MSA subjects via the individual PCA expression scores. To confirm presence or absence of systematic differences between scanner types and sites that could bias the PCA expression score, we compared all subgroups divided by centre and scanner type. To this end, we used unpaired *t*-tests for all PCA expression *z*-scores and corrected the resulting *p*-values for multiple testing using false discovery rate (FDR) correction.

## Results

### Demographics

Demographics and clinical parameters of the DLB subgroups and comparison between groups are shown in Table 1. The disease duration was defined as the elapsed time between the first symptoms and date of FDG-PET scanning. Among the overall 108 patients with sufficient background and imaging data, 86 subjects showed a significant dopaminergic deficiency, whereas 22 subjects evinced no significant pathologic result in their DaT-SPECT scan (*z*-score < 2 in DaT-SPECT compared to healthy controls). In the DLB-DaT(+) group, 77% received the diagnosis of probable DLB according to the McKeith criteria. In the DLB-DaT(−) group, the percentage of patients with probable DLB diagnosis was 68%. Rapid eye movement sleep behaviour disorder was less frequent in DLB-DaT(−) when compared to DLB-DaT(+) (17% vs. 44%, *p* = 0.034).

**Table 1:** Demographics of the DLB cohort: Patients with DLB of this multicentre dataset were divided into a subgroup of individuals with dopaminergic deficiency (DLB-DaT(+)) and a subgroup of individuals with preserved dopaminergic function (DLB-DaT(−)). The categorization was based on the *z*-score of putaminal DaT availability. *n*, number; *MMSE*, Mini Mental Status Examination; age refers to the age at the time of FDG-PET. *RBD*, rapid eye movement sleep behaviour disorder

	All	DaT(+)	DaT(−)	DaT(+) vs. DaT(−)
*N*	108	86	22	
Age (FDG-PET)	72.9 ± 7.5	72.8 ± 7.7	73.5 ± 6.8	*p* = 0.677
Sex	♂ 66 ♀ 42	♂ 54 ♀ 32	♂ 12 ♀ 10	*p* = 0.625
Education (y, *n* = 102)	12.3 ± 3.5	12.2 ± 3.5	12.9 ± 3.6	*p* = 0.469
Disease duration (y, *n* = 99)	2.7 (0.3 to 10.1)	2.8 (0.4 to 9.0)	2.5 (0.3 to 10.1)	*p* = 0.631
MMSE (0–30, *n* = 74)	22.5 ± 4.8	22.6 ± 4.8	22.0 ± 4.8	*p* = 0.665
Probable/possible DLB (%)	75/25	77/23	68/32	*p* = 0.203
Parkinsonism (%)	84	83	86	*p* = 1.000
Visual hallucinations (%)	57	55	62	*p* = 0.631
Fluctuating cognition (%)	69	69	67	*p* = 1.000
RBD (%)	39	44	17	*p* = 0.034
Putaminal DaT availability (*z*-score)	− 3.2 ± 1.3 (− 6.14 to + 0.45)	− 3.7 ± 0.9 (− 6.14 to − 2.0)	− 1.1 ± 0.7 (− 1.98 to + 0.45)	*p* < 0.001

### FDG-PET glucose metabolism pattern in DLB-DaT(+) and DLB-DaT(−)

**Fig. 2:**
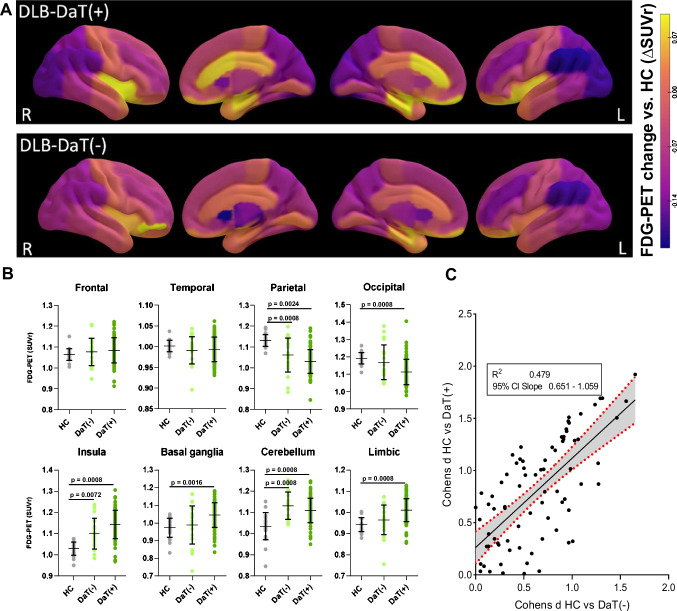
Glucose uptake in comparison of DLB-DaT(+) and DLB-DaT(−). **A** Surface projections of global mean scaled FDG-PET SUVr changes for both DLB cohorts compared with the HC group. Glucose hypometabolism in the parieto-occipital cortices was more prominent but regionally similar in patients with manifest dopamine deficiency (DLB-DaT(+)) compared to those with preserved dopamine transmission (DLB-DaT(−)). Similar hypermetabolism patterns were observed in the motor cortex as well as basal ganglia and limbic system. A supplemental voxel-based analysis is provided in Supplemental Fig. 1. **B** Individual values of global mean scaled FDG-PET (SUVrs) depicting the metabolic changes in eight composite regions by comparing DLB-DaT(+), DLB-DaT(−) and HCs. *p*-values are shown after Bonferroni correction. No significant changes were observed for direct comparisons between DLB-DaT(+) and DLB-DaT(−). Individual subject categorization by altered regional global mean scaled FDG-PET SUVr is shown in Supplemental Fig. 2. **C** Correlation of regional metabolic changes (Cohen’s *d*) in 77 brain regions between DLB-DaT(+) and DLB-DaT(−). Cohen’s *d* were calculated for global mean scaled FDG-PET SUVr for DLB-DaT(+) vs HC and DLB-DaT(−) vs HC

Compared to HC, DLB-DaT(+) patients showed the expected relative reduction in glucose metabolism in parieto-occipital and frontal cortices (superior, middle, inferior and orbitofrontal), whereas a relative glucose hypermetabolism was observed in motor cortices, the basal ganglia, parts of the limbic system and the cerebellum (Fig. 2A). Precuneus and posterior cingulate cortex showed no significant glucose alterations. DLB-DaT(−) patients expressed similar, overall less pronounced, patterns of relative hypo- and hypermetabolism. Single region global mean scaled FDG-PET SUVr values of all three groups are provided in Supplemental Table 4.

Compared to DLB-DaT(−), the DLB-DaT(+) patients showed a more distinct relative hypometabolism in above-mentioned occipital and basal structures of the brain (Fig. 2B). The insula exhibited a more prominent hypermetabolism for those subjects whose DaT-SPECT showed dopamine deficiency. Effect sizes of single region alterations in DLB vs. controls were strongly correlated between DLB-DaT(−) and DLB-DaT(+) patients (*R*^2^ = 0.479, *p* < 0.0001; Fig. 2C).

### Metabolic connectivity in DLB-DaT(+) and DLB-DaT(−)

**Fig. 3:**
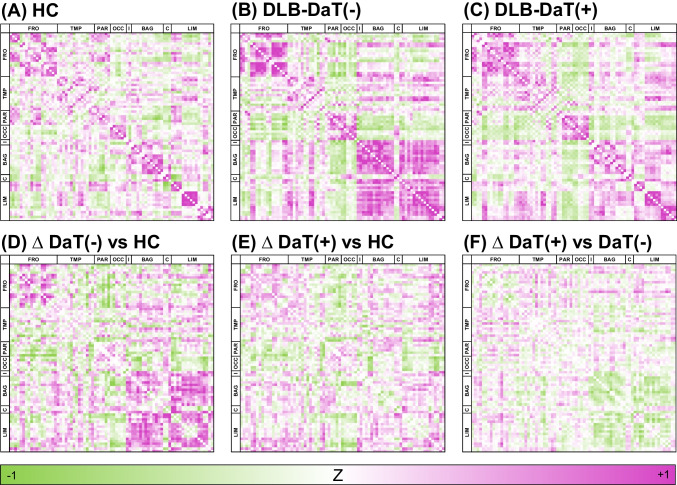
Metabolic connectivity patterns in DLB-DaT(+), DLB-DaT(−) and healthy controls (HC). **A**–**C** Group-level inter-region correlation coefficients (ICC) after Fisher transformation are shown per region pair (77 × 77) for HC and DLB patients without significant dopamine deficiency (DaT(−)) and DLB patients with pathological DaT-SPECT (DaT(+)). **D**–**F** Differences (delta = ∆) of ICC, comparing DLB-DaT(+) with HC, DLB-DaT(−) with HC and DLB-DaT(+) with DLB-DaT(−) patients. Purple indicates positive ICCs whereas green indicates negative ICCs

**Fig. 4:**
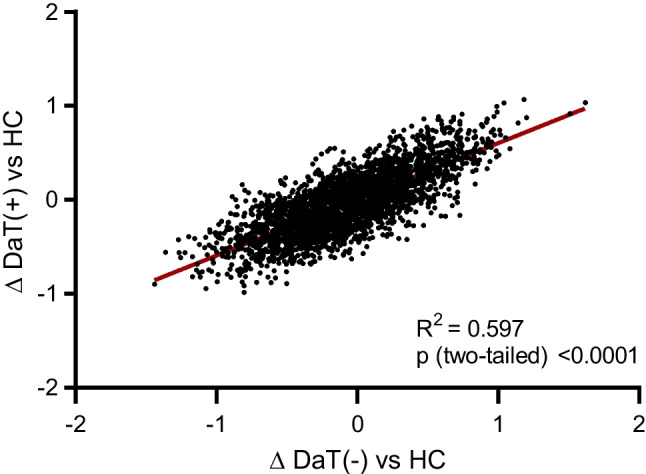
Similarity analysis of metabolic connectivity patterns. Differences of regional ICC in DLB-DaT(+) patients compared to healthy controls were plotted against differences of regional ICC in DLB-DaT(−) patients compared to healthy controls as linear regression model. ICC, inter-region correlation coefficients

Metabolic connectivity was analysed in both DLB subgroups (DLB-DaT(+)/DLB-DaT(−)) and compared to the control group (23 subjects) with presumably intact nigrostriatal dopamine innervation (Fig. 3). In line with our recent publication [18], the most prominent increase in metabolic connectivity of DLB patients occurred within the basal ganglia, frontal cortices and limbic system as well as between limbic system and basal ganglia [18]. Although some brain regions indicated a different magnitude of metabolic alteration between DLB-DaT(+) and DLB-DaT(−) patients, the overall regional connectivity changes were strongly associated between DLB-DaT(+) and DLB-DaT(−) patients (*R*^2^ = 0.597, *p* < 0.0001; Fig. 4). Single region metabolic connectivity values are provided in Supplemental Table 4.

### Single subject categorization

**Fig. 5:**
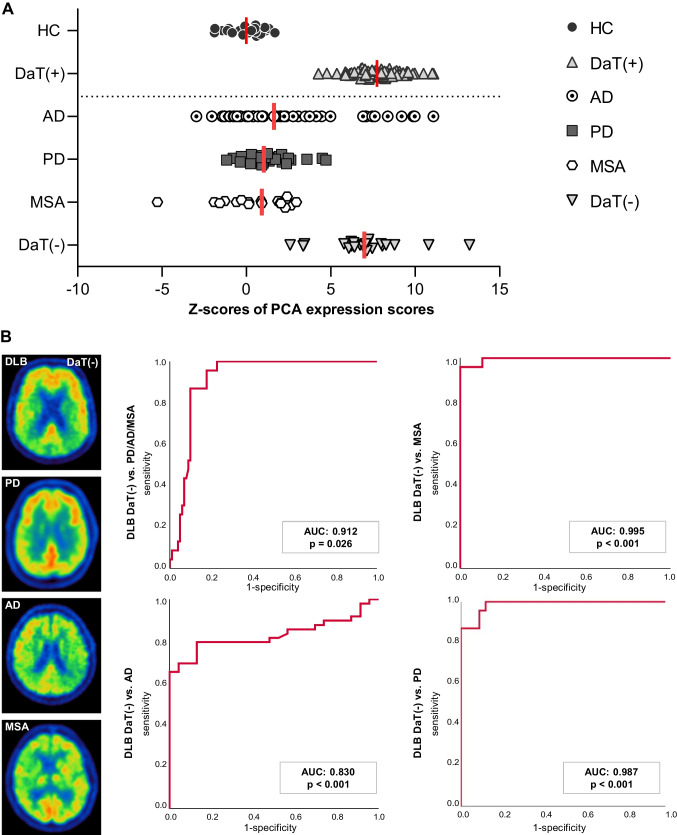
Principal component analysis for discrimination of DLB-DaT(−) patients from other neurodegenerative diseases. **A** *z*-scores distribution of PCA expression scores (relative to healthy controls, HC) for the different groups of patients with neurodegenerative diseases. PCA was trained by the comparison of DLB-DaT(+) patients and HC (data above the dotted line). The PCA expression *z*-scores for individual DLB-DaT(−) patients differed considerably from patients with other neurodegenerative diseases and showed strong agreement with the *z*-scores of DLB-DaT(+) subjects. **B** ROC analyses show areas under the curve (AUC) for discrimination between DLB DaT(−) and the subgroups of differential diagnoses. Left column shows individual FDG-PET results of the respective diagnosis groups. PCA, principal component analysis; MSA, multiple system atrophy; PD, Parkinson’s disease; AD, Alzheimer’s disease

Given the similarity of metabolic connectivity alterations in DLB-DaT(+) and DLB-DaT(−) group, we hypothesized that a data-driven network analysis could facilitate the identification and discrimination of DLB-DaT(−) patients against other neurodegenerative diseases. In order to implement the findings of this study into clinical scenarios, we conducted a PCA on the basis of regional FDG-PET values of the established cohort. We trained the PCA by DLB-DaT(+) patients and controls (Supplemental Fig. 3A) and derived individual expression scores from individual patients of a test cohort including DLB-DaT(−), AD, MSA and PD. The overall variance of PCA expression scores for controls of the training cohort was low (CoV 16.4%). Significant floating of the PCA by adding additional single subjects was excluded by a robust coefficient of variation (7.4%) in the PCA expression scores of controls. PCA expression *z*-scores of DLB-DaT(−) patients were higher (6.97 ± 2.29) when compared to PCA expression *z*-scores of other neurodegenerative diseases (MSA: 0.63 ± 2.10; PD: 1.27 ± 1.40; AD: 2.42 ± 3.58; Fig. 5A). The magnitude of PCA expression scores in DLB-DaT(−) patients was similar compared to DLB-DaT(+) patients (Supplemental Fig. 3B). We then conducted separate ROC analyses for the DLB-DaT(−) scores against the scores of the other conditions. ROC curves indicated excellent discrimination of DLB-DaT(−) patients against the whole cohort of degenerative diseases (AUC: 0.912; Fig. 5B). As expected, discriminatory power was highest for the comparison of DLB-DaT(−) against PD (AUC: 0.995) and MSA (AUC: 0.987), and still at a high level for the comparison of DLB-DaT(−) against AD (0.830). Discrimination of DLB-DaT(−) patients by PCA expression scores was stronger compared to discrimination by regional global mean scaled FDG-PET SUVr values (Supplemental Fig. 4). PCA expression scores of the whole DLB cohort correlated weakly but significant with cognitive screening (MMSE; *p* = 0.019, *R*^2^ = 0.073), indicating stronger correlation for the DLB-DaT(+) subjects (*p* = 0.002, *R*^2^ = 0.160; Supplemental Fig. 5A). The correlation between DaT availability (putaminal *z*-score) and PCA expression scores was close to significance for the entire DLB-cohort, driven by a substantial correlation in DLB-DaT(−) patients (all DLB: *p* = 0.050, *R*^2^ = 0.036; DLB-DaT(−): *p* = 0.029, *R*^2^ = 0.216; Supplemental Fig. 5B). PCA expression scores were not significantly different in presence or absence of clinical core features (all *p* > 0.200, Supplemental Fig. 5C).

## Discussion

The present study suggests that metabolic connectivity alterations can serve as a supporting biomarker for the diagnosis of suspected DLB without significant dopaminergic loss. Metabolic changes and connectivity patterns of DLB patients without dopaminergic deficiency strongly correlate with the respective alterations already known for DLB patients with manifest dopaminergic loss. Furthermore, a metabolic connectivity pattern PCA expression score successfully differentiated DLB patients without significant dopaminergic loss from other neurodegenerative diseases and could aid as a supporting biomarker in clinical decision-making.

Glucose hypometabolism especially in parieto-occipital cortices as well as prominent hypermetabolism in motor cortices, basal ganglia and limbic system was confirmed for DLB patients without significant dopaminergic loss (Fig. 2). These alterations of glucose metabolism were well in line with the patterns observed in DLB patients with significant dopaminergic loss and previously reported patterns of glucose metabolism alterations in DLB [31–33]. However, significant changes of glucose metabolism levels (≥ 2 SD of controls) were only obvious in 68% of DLB-DaT(−) patients but in 79% of DLB-DaT(+) patients (Supplemental Fig. 2) which also translated into moderate AUCs in the ROC analysis for the discrimination of DLB-DaT(−) against other neurodegenerative diseases (Supplemental Fig. 4). Therefore, we interrogated the value of FDG-PET to detect of DLB with minimal dopamine transporter degeneration. Relative hypermetabolism in basal ganglia and limbic system was already present, though less pronounced, for DLB-DaT(−) subjects [18, 34, 35]. Thus, we asked whether a network-based analysis would outperform a ROI-based evaluation.

Past research has extensively examined the relationship between Parkinson’s disease-related pattern (PDRP) network expression and dopamine deficiency [36, 37]. In DLB, dopaminergic loss correlates with the characteristic metabolic patterns that have been shown to support diagnosis. While metabolic connectivity increases with slight dopaminergic loss in these regions, there is a decline in connectivity in DLB-DaT(+) subjects, possibly due to advanced disease stages leading to a multi-regional neuronal degeneration. Other areas, specifically the parieto-occipital cortex and the connection between parieto-occipital and limbic brain structures, indicated a uniform decrease in metabolic connectivity levels, potentially following the disease spread [18]. Deep learning-based analysis of FDG-PET even predicted DLB at 96% accuracy when combining two large multi-centre databases [38]. Thus, we questioned whether metabolic connectivity alterations were similar between DLB-DaT( −) and DLB-DaT(+) patients. Interestingly, we observed that DLB patients without significant dopaminergic loss had prominent alterations in metabolic connectivity, occurring in the same interregional linkages that also showed metabolic pattern changes in DLB patients with manifest dopaminergic loss at the global brain level (Fig. 3). Regions of hypometabolism also displayed reduced connectivity levels, whereas connectivity increased in regions with elevated glucose metabolism in DaT(−) subjects. These findings are in line with the compensatory recruitment hypothesis, proposing that connectivity may initially increase in early stages of the disease with new brain areas being recruited to compensate for degenerating regions, while later disease stages lead to the collapse of these compensatory mechanisms. Previous studies have shown similar results using other biomarkers such as fMRI [39, 40] or dopaminergic imaging [41]. Our data cannot answer the question if DLB-DaT(−) patients represent an early stage of a DLB continuum or a distinct DLB phenotype. However, we note that DLB-DaT(−) patients had a statistically lower frequency of REM sleep behaviour disorder, which could imply a phenotypical difference to the group with pathological DaT-SPECT findings, potentially due to a different neurodegenerative spread of α-synuclein. Thus, similar metabolic connectivity alterations may be the joint feature of both phenotypically distinct subgroups.

Our results support the use of metabolic connectivity alterations to diagnose DLB patients without significant dopamine deficiency, since discrimination against other neurodegenerative disorders was also feasible at the individual patient level. The presented single-patient PCA approach can be implemented in routine software packages for analysis of FDG-PET with moderate effort, paving the way for the establishment of a clinically applicable biomarker for early DLB diagnosis. The applied PCA approach is highly similar to SSM/PCA strategies [21, 42], likewise using the principal components in a mixed cohort of patients with DLB and controls. Both approaches transfer the principal components to a regression model in order to determine which factors discriminate best between patients with DLB and controls. As a consequence, the obtained DLB related pattern (Supplemental Fig. 3A) was similar to previously SSM/PCA strategies [21, 42]. We provide the source files of our training set and the expression score calculation attached to the manuscript to allow determination of single-patient DLB probability by simple assessment of Hammers atlas global mean scaled SUVr. Discrimination between DLB and AD is often more difficult due to considerable regional overlap of affected brain regions in those two disease entities [43]. Therefore, a lower AUC was observed for differentiation between DLB-DaT(−) patients from AD, while still providing good discriminatory accuracy. Our data are in line with a FDG-PET study that stratified the prodromal stage of DLB by clinical symptoms (MCI) [32]. Here, a medial temporal to substantia nigra ratio distinguished MCI-DLB from MCI-AD at high sensitivity and specificity [32].

## Limitations

A main limitation of this study consists in the lack of histopathological diagnostic confirmation of diagnosis as well as systematic evaluation of CSF biomarkers. Therefore, we cannot exclude the possibility of misdiagnosis for some cases. However, we used the latest diagnostic criteria for possible and probable DLB and clinical follow-up was ensured [8]. As the DLB subjects in our cohort underwent both FDG-PET and DaT-SPECT imaging, a potential selection bias might occur in the sense of a more complex subject group of DLB patients compared to the general population. Other potential confounders include the clinical examination processes since diagnoses were made by different clinical experts and in different clinical settings which might have introduced bias. However, the latest McKeith diagnostic criteria were applied uniformly. Additionally, while the two subgroups had similar overall frequency of parkinsonism, severity of parkinsonism was not consistently assessed by UPDRS motor scale. Thus, severity of parkinsonism may act as a potential correlative index for dopamine deficiency in this DLB cohort and could not be accounted for. FDG-PET data was filtered and normalized to global mean to minimize the mismatch between post-processing among different centres, but some residual influence caused by the multicentre approach of this study likely remained. Supplemental Table 5A to D show FDR-corrected *p*-values and effect sizes for all scanner and site specific comparisons of PCA *z*-scores including the main cohorts (HC, DLB total, DLB-DaT(+) and DLB-DaT(−)). There were no significant differences between PCA expression *z*-scores of different sites and PET scanners.

Moreover, global mean normalization could potentially generate an artificial heightening of glucose metabolism levels caused by the disease. To cope with effects of different sites and scanners, we used a robust VOI-based analysis, but we note that a voxel-based approach could be more sensitive to metabolic differences between DLB subgroups with and without significant dopaminergic loss. Finally, while the cut-off of  − 2 *z*-score has been widely used for definition of abnormal DaT-SPECT, less conservative cut-offs have been identified in previous studies both for prodromal and later stages of DLB [44, 45].

## Conclusion

Our data indicate that disease-specific patterns of altered glucose metabolism and altered metabolic networks are present in DLB subjects without significant dopaminergic loss. Metabolic network alterations in FDG-PET have a potential as supporting biomarker for DLB-DaT(−) and warrant validation in prospective studies with long-term clinical follow-up or post-mortem validation.

### Supplementary Information

Below is the link to the electronic supplementary material.

Supplementary file1 (DOCX 1.52 MB)

Supplementary file2 (DOCX 47 KB)

Supplementary file3 (XLSX 225 KB)

## Data Availability

The datasets generated during and/or analysed during the current study are available from the corresponding author on reasonable request.
