# Supplementary material for: Metabolic network alterations as a supportive biomarker in dementia with Lewy bodies with preserved dopamine transmission
Source: Eur J Nucl Med Mol Imaging. 2023 Nov 16;51(4):1023–34. doi: 10.1007/s00259-023-06493-w (PMC10881642; doi:10.1007/s00259-023-06493-w)
Supplement: Supplementary file 1 — Supplementary file1 (DOCX 1.52 MB) [file 259_2023_6493_MOESM1_ESM.docx]

**Supplement to: Metabolic network alterations as a supportive biomarker in Dementia with Lewy Bodies with preserved dopamine transmission**

**Supplemental figures**

**Supplemental Figure 1 – Voxel-wise comparison**


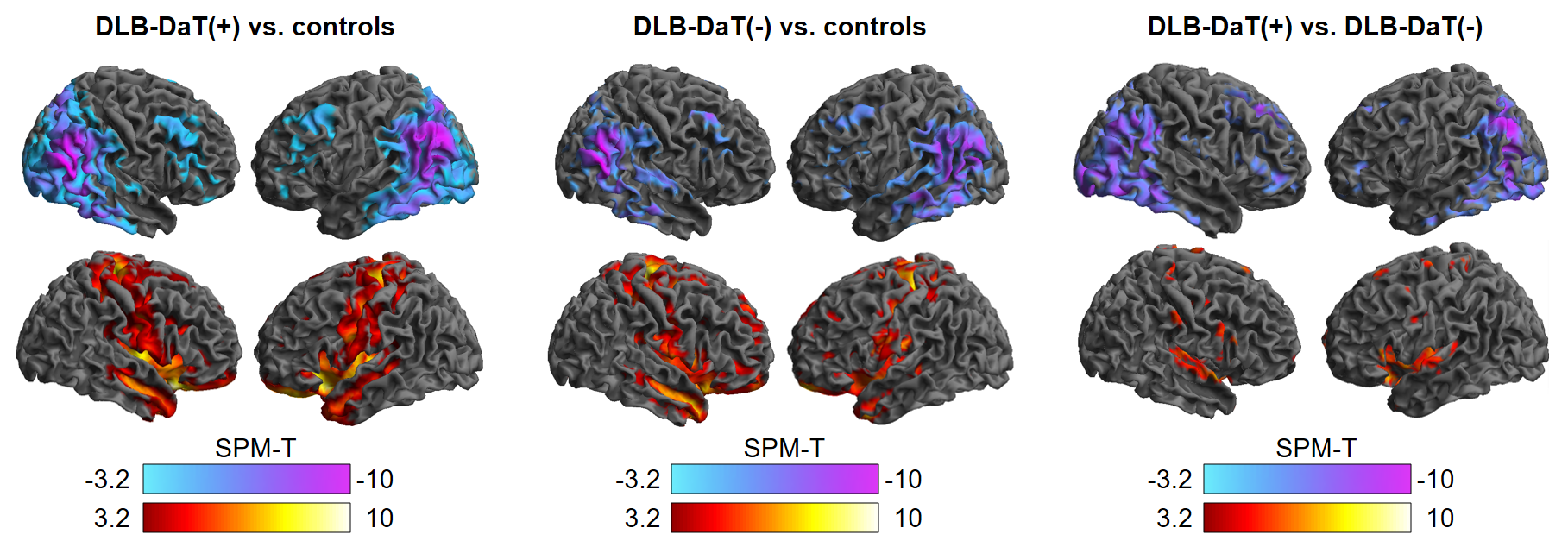


**Supplemental Figure 1** Voxel-wise comparison of both DLB groups and controls. Surface projections provide significant voxels at a p-level of 0.001 uncorrected for multiple comparisons. Cluster threshold: 50 voxel.

**Supplemental Figure 2 – Single region performance**


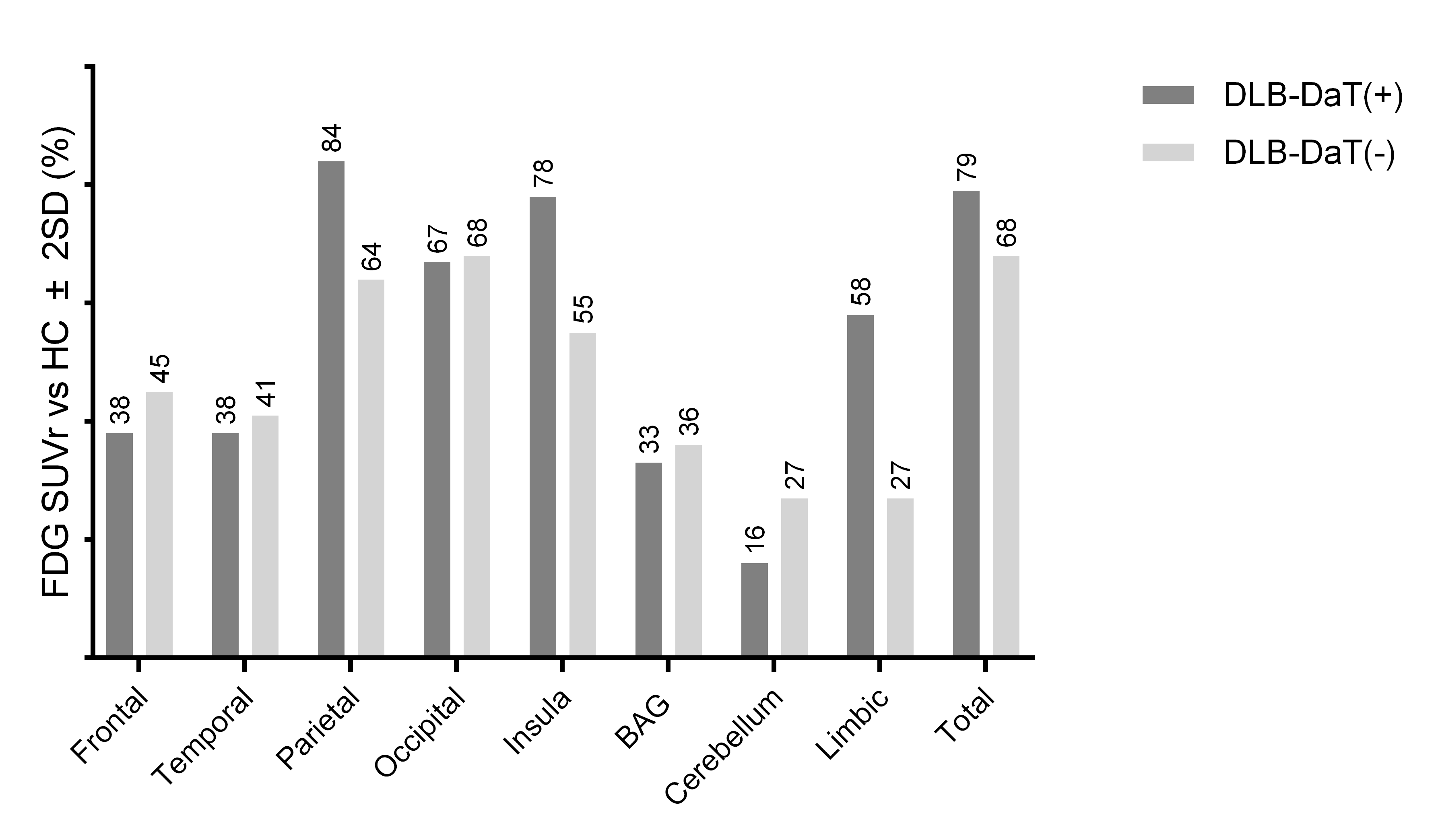


**Supplemental Figure 2** Percentage of significant glucose metabolism alterations ± 2SD per region. Significant alteration of glucose metabolism was defined as a deviation of ≥ 2 SD of HC. In the DLB-DaT(-) cohort 68% of the patients reached this cut-off, compared to 79% of DLB-DaT(+) patients.

**Supplemental Figure 3 – Principal components**


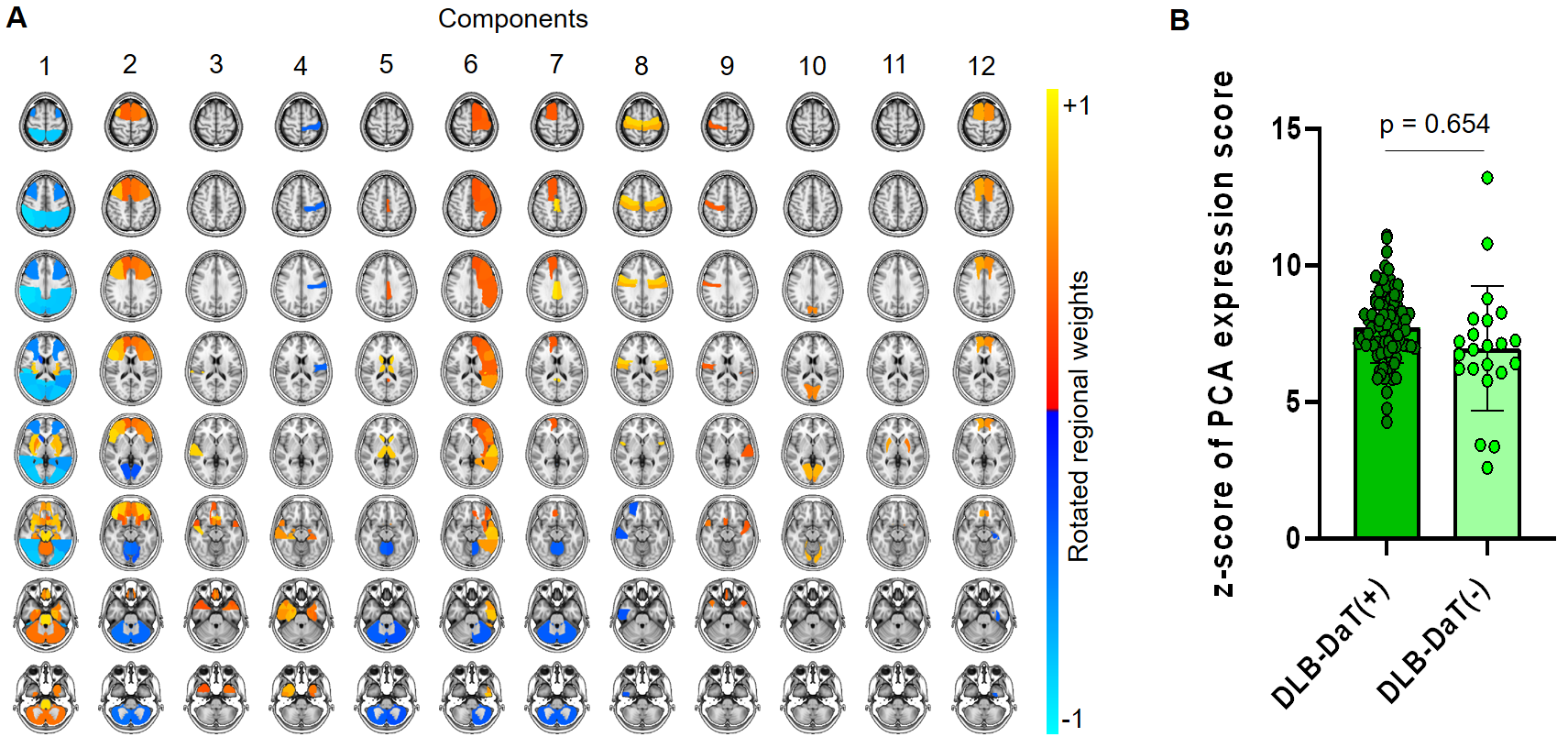


**Supplemental Figure 3** (**A**) Rotated regional weights of [^18^F]FDG SUVr z-score components identified by applying principal component analysis on 77 cerebral regions. The colors represent the region-specific weights (range from -1 to 1) on each component. (**B**) Direct comparison of the PCA expression z-scores (relative to healthy controls) of DLB-DaT(+) patients (derived from the training cohort) and DLB-DaT(-) patients (derived from PCA test runs). P value derives from one-way ANOVA of Fig. 5A group comparison.

**Supplemental Figure 4 – Single region ROC**


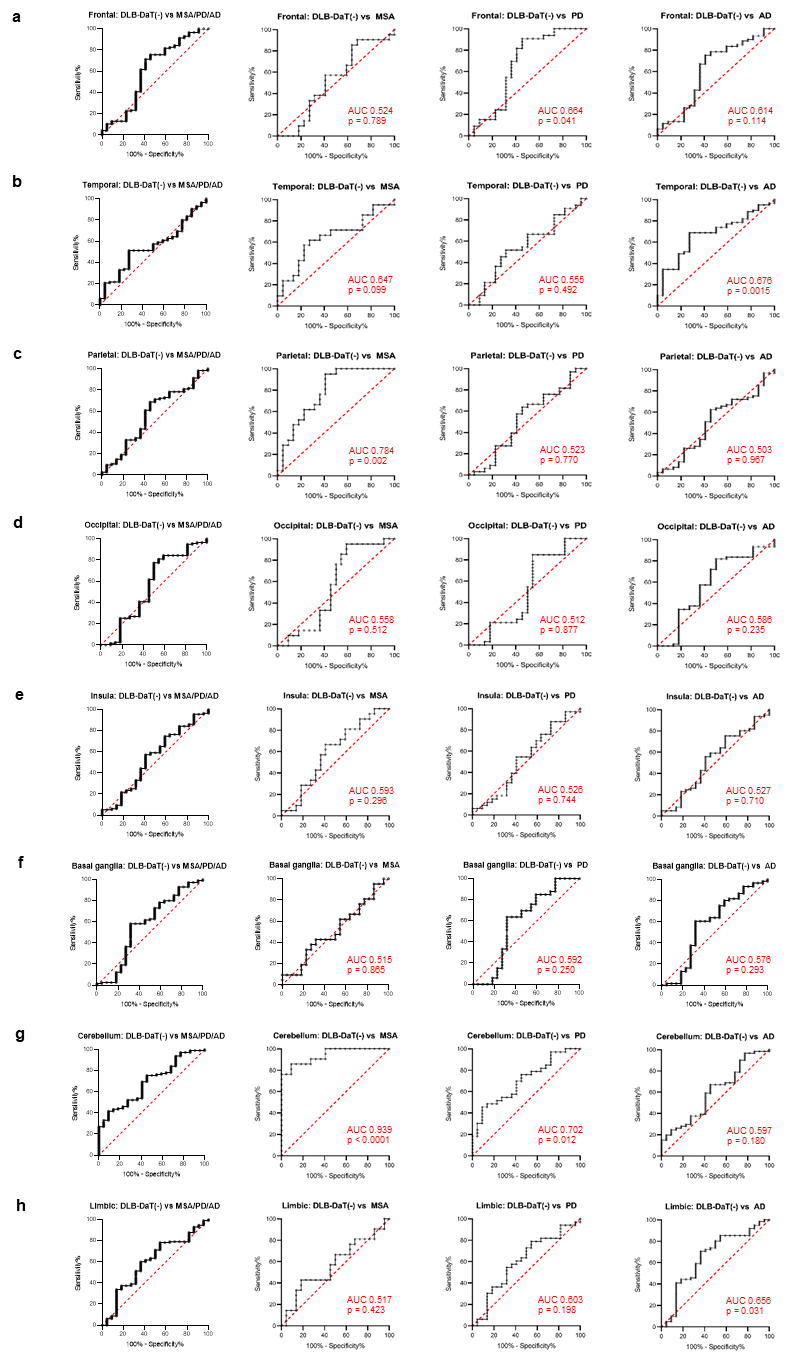


**Supplemental Figure 4** AUC of regional SUVrs for DLB-DaT(-) vs other neurodegenerative differential diagnoses. Regional SUVr perform rather weakly as discriminating factor for DLB-DaT(-) patients against other neurodegenerative disorders.

**Supplemental Figure 5 – Clinical and biomarker correlation of PCA z-scores**

**
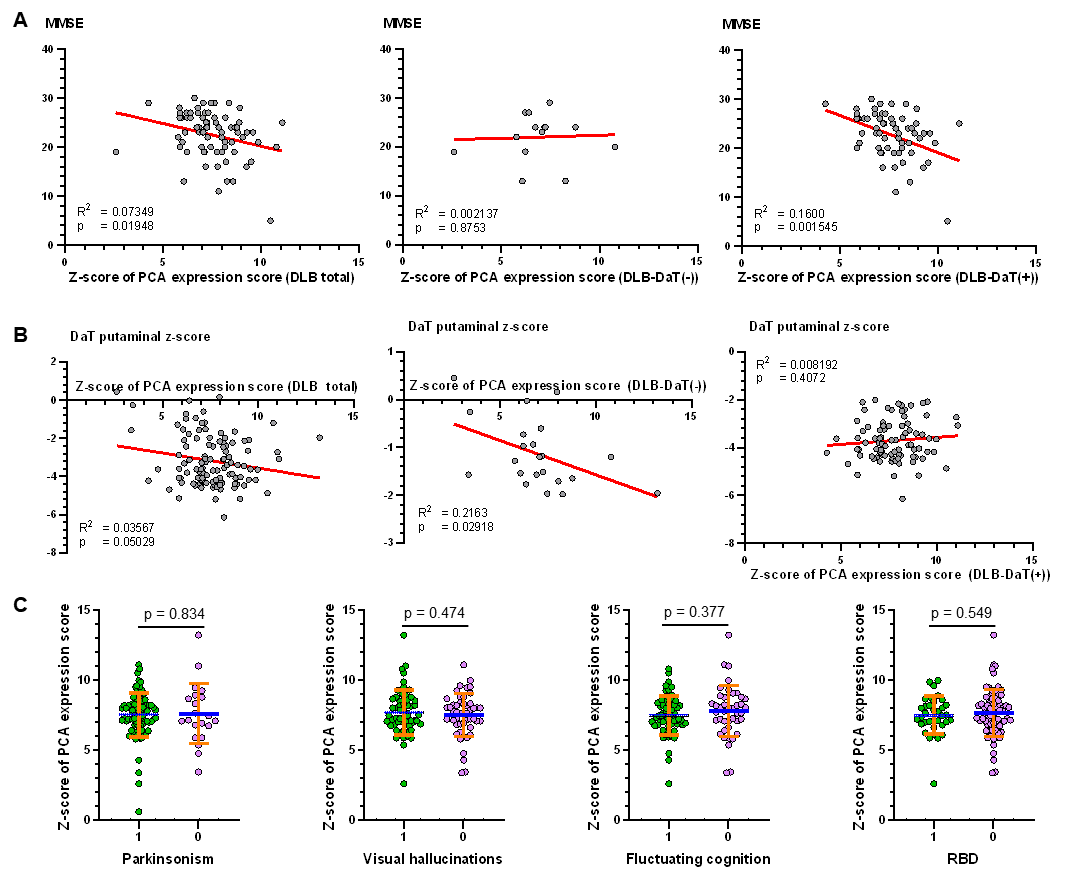
**

**Supplemental Figure 5** Correlations of clinical features with z-scores of the principal component analysis (PCA). MMSE and dichotomous DLB core features as well as quantitative dopamine transporter availability were available for correlation with z-scores of PCA pattern expression within the DLB cohort. (**A**) Correlation between MMSE scores and the PCA expression z-score for the entire DLB cohort, DLB-DaT(-) and DLB-DaT(+) (left to right) as well as (**B**) respective correlations for putaminal DaT-SPECT z-scores. (**C**) PCA expression z-scores in comparison of the presence or absence of dichotomous clinical core features of DLB (Parkinsonism, visual hallucinations, fluctuating cognition, REM-sleep behavior disorder / RBD).

**Supplemental tables**

**Supplemental Table 1 - Demographics**

|  | HC (FDG-PET) |
| --- | --- |
| N | 23 |
| Age (y, At fdg-pet) | 74.5 ± 6.0 |
| Sex | ♂ 9 ♀ 14 |
|  |  |
|  | **HC (DaT-SPECT)** |
| N | 37 |
| Age (y, at DaT-spect) | 70.1 ± 6.0 |
| Sex | ♂ 21 ♀ 16 |
|  |  |
|  | **PD** |
| N | 34 |
| Age (y, at FDG-PET) | 72.9 ± 9.5 |
| Sex | ♂ 21 ♀ 13 |
| Age at symptom onset (n=18) | 66.0 ± 12.1 |
| H&Y (n= 21) | Stage 1: n=10; Stage 2: n=7; Stage 3: n=4 |
| Symptom duration (y) | 3.7 ± 2.7 |
| Clinical PHENOTYPE | PD n=25; PDD n=9 |
|  |  |
|  | **AD** |
| N | 49 |
| Age (y, at FDG-PET) | 68.9 ± 8.9 |
| Sex | ♂ 23 ♀ 26 |
| MMSE (0-30, n=4574) | 22.3 ± 5.5 |
| Aß-Status (PET) | positive: n= 49 |
| Clinical PHENOTYPE | MCI / prodromal AD n=21; AD dementia  n=16; PPA n=2; PCA n=2; mixed clinical phenotype with positive AD biomarker status n=8 |
|  |  |
|  | **MSA** |
| N | 19 |
| Age (y, at FDG-PET) | 63.8 ± 9.0 |
| Sex | ♂ 10♀ 9 |
| Subtype | Cerebellar 8 , Parkinsonian 10, mixed 1 |
| H&Y (n= 16) | Stage 1: n=2; Stage 2: n=9; Stage 3: n=4; Stage 5: n=1 |
| Symptom duration (y) | 2.6 ± 2.1 |
| Clinical confidence | probable n=13, possible n=6 |
| UPDRS (n= 11) | 33.7 ± 12.3 |
|  |  |

**Supplemental Table 1** Demographics of the cohorts of HCs and other neurodegenerative diseases (PD, AD, MSA). HC = Healthy controls; PD = Parkinson’s disease; PDD = Parkinson’s disease dementia; AD = Alzheimer’s disease; MSA = Multiple systems atrophy; PPA = Primary progressive aphasia; PCA = Posterior cortical atrophy; Y = Age in years; H&Y = Hoehn & Yahr stage; UPDRS = Unified Parkinson’s Disease Rating Scale; MMSE = Mini Mental Status Exam; MCI = mild cognitive impairment; Aβ-status = amyloid-beta status.

**Supplemental Table 2 – Overview on sites and scanners**

| Site | subjects (n, DLB/HC) | | SPECT CAMERA | Scanner DETAILS | PET Scanner |
| --- | --- | --- | --- | --- | --- |
| MUNICH | 47/9 | Prism 3000, Picker, Cleveland, OH | | triple-head gamma camera | Siemens Biograph 64  Siemens Biograph mCT (16 slice CT)  GE Discovery 710 PET/CT |
| Genova | 15/14 | Discovery®, G.E. Healthcare, Hatfeld, Hertfordshire, UK | | dual-head, parallel-hole, high-resolution collimator camera | Siemens Biograph mCT (16 slice CT) |
| Geneva | 14 | GCA-9300A/UI Toshiba Medical Systems AG, Oetwil am See, Switzerland | | triple-head gamma camera | Siemens Biograph mCT (128 slice CT) |
| Ljubljana | 7 | Siemens Symbia T2 | | dual-head gamma camera | Siemens Biograph mCT (128 slice CT) |
| Linkoping | 12 | Millenium VG with low-energy, high-resolution (LEAR) collimator, General Electric | | dual-head gamma camera | GE Discovery 710 PET/CT |
| Stockholm | 4 | Siemens Symbia T16 | | dual-head gamma camera | Biograph mCT (Siemens) |
| Leuven | 9 | Discovery®, G.E. Healthcare, Hatfeld, Hertfordshire, UK | | dual-head, parallel-hole, high-resolution collimator camera | Siemens Biograph (16 slice CT) |

**Supplemental Table 2** Details of the seven European centers where imaging data for the DLB and HC cohorts were acquired via the E-DLB Consortium. Munich and Genova were the two centers providing the healthy control data.

**Supplemental Table 3 – Linear regression of principal components**

|  | Non standardized coefficients | | Stand. coefficients |  | |  | 95% CI | |
| --- | --- | --- | --- | --- | --- | --- | --- | --- |
|  | regression coefficient | standard error | beta (β) | T | | p-value | lower CI threshold | upper CI threshold |
| REGR factor score 1 | 0.306 | 0.015 | 0.748 | 20.083 | 0.000 | | 0.276 | 0.337 |
| REGR factor score 2 | -0.027 | 0.015 | -0.066 | -1.765 | 0.081 | | -0.057 | 0.003 |
| REGR factor score 3 | -0.008 | 0.015 | -0.019 | -0.512 | 0.610 | | -0.038 | 0.022 |
| REGR factor score 4 | 0.019 | 0.015 | 0.047 | 1.249 | 0.215 | | -0.011 | 0.049 |
| REGR factor score 5 | -0.107 | 0.015 | -0.260 | -6.995 | 0.000 | | -0.137 | -0.076 |
| REGR factor score 6 | -0.080 | 0.015 | -0.196 | -5.253 | 0.000 | | -0.110 | -0.050 |
| REGR factor score 7 | 0.104 | 0.015 | 0.253 | 6.809 | 0.000 | | 0.074 | 0.134 |
| REGR factor score 8 | -0.049 | 0.015 | -0.120 | -3.231 | 0.002 | | -0.080 | -0.019 |
| REGR factor score 9 | -0.010 | 0.015 | -0.024 | -0.633 | 0.528 | | -0.040 | 0.021 |
| REGR factor score 10 | 0.010 | 0.015 | 0.025 | 0.662 | 0.509 | | -0.020 | 0.040 |
| REGR factor scorE 11 | -0.129 | 0.015 | -0.315 | -8.450 | 0.000 | | -0.159 | -0.099 |
| REGR factor score 12 | -0.052 | 0.015 | -0.127 | -3.422 | 0.001 | | -0.083 | -0.022 |

**Supplemental Table 3** Linear regression of principal components of the training set consisting of healthy controls (HC) and patients with disease with Lewy bodies and dopaminergic loss (DLB-DaT(+)). Disease status (DLB vs. HC) was used as outcome variable. Standardized regression coefficients (β) were multiplied with respective PCs as weighting factors before calculating the expression score as a sum of all PCs per individual subject.

**Supplemental Table 4** **– Average FDG-PET values and metabolic connectivity values**

| Hammers Region | HC mean SUVr | DLB-DaT(-) mean SUVr | DLB-DaT(+) mean SUVr | HC mean MC (abs) | DLB-DaT(-) mean MC (abs) | DLB-DaT(+) mean MC (abs) |
| --- | --- | --- | --- | --- | --- | --- |
| FL_mid_fr_G_l | 1.0965 | 0.9588 | 0.9707 | 0.3921 | 0.3431 | 0.3104 |
| FL_mid_fr_G_r | 1.1445 | 1.0724 | 1.0621 | 0.3424 | 0.3385 | 0.2763 |
| FL_precen_G_l | 1.0564 | 1.0154 | 1.0309 | 0.2998 | 0.2323 | 0.2121 |
| FL_precen_G_r | 1.0985 | 1.0845 | 1.0937 | 0.2833 | 0.3187 | 0.1546 |
| FL_strai_G_l | 1.0135 | 1.0908 | 1.1149 | 0.2488 | 0.4571 | 0.4239 |
| FL_strai_G_r | 1.0090 | 1.0546 | 1.0892 | 0.1698 | 0.4282 | 0.3988 |
| FL_OFC_AOG_l | 1.0744 | 1.1120 | 1.1111 | 0.2263 | 0.3496 | 0.2680 |
| FL_OFC_AOG_r | 1.0805 | 1.0966 | 1.0834 | 0.2924 | 0.3465 | 0.2442 |
| FL_inf_fr_G_l | 1.0827 | 1.0149 | 1.0374 | 0.3366 | 0.3601 | 0.2815 |
| FL_inf_fr_G_r | 1.1375 | 1.1212 | 1.1196 | 0.2828 | 0.2830 | 0.2559 |
| FL_sup_fr_G_l | 1.0187 | 0.9469 | 0.9572 | 0.3569 | 0.3241 | 0.2749 |
| FL_sup_fr_G_r | 1.0134 | 0.9605 | 0.9713 | 0.3429 | 0.3551 | 0.2504 |
| FL_OFC_MOG_l | 1.0365 | 1.1216 | 1.1353 | 0.3347 | 0.3738 | 0.3504 |
| FL_OFC_MOG_r | 1.0491 | 1.1009 | 1.1254 | 0.2758 | 0.3559 | 0.3822 |
| FL_OFC_LOG_l | 1.0749 | 1.0793 | 1.0780 | 0.2234 | 0.3523 | 0.2859 |
| FL_OFC_LOG_r | 1.1557 | 1.1727 | 1.1594 | 0.2592 | 0.3077 | 0.2624 |
| FL_OFC_POG_l | 1.0228 | 1.0922 | 1.1224 | 0.2460 | 0.4208 | 0.3887 |
| FL_OFC_POG_r | 1.0309 | 1.0899 | 1.1146 | 0.2282 | 0.4432 | 0.3662 |
| Ant_TL_med_r | 0.7603 | 0.7753 | 0.8101 | 0.1900 | 0.3483 | 0.2807 |
| Ant_TL_med_l | 0.7974 | 0.8503 | 0.8711 | 0.2719 | 0.3386 | 0.3104 |
| Ant_TL_inf_lat_r | 0.9117 | 0.9136 | 0.9225 | 0.2073 | 0.1732 | 0.3059 |
| Ant_TL_inf_lat_l | 0.9380 | 0.9348 | 0.9552 | 0.2350 | 0.3086 | 0.2775 |
| G_sup_temp_post_r | 1.0716 | 1.0451 | 1.0805 | 0.1780 | 0.2242 | 0.2311 |
| G_sup_temp_post_l | 1.0640 | 1.0167 | 1.0556 | 0.2957 | 0.2997 | 0.1930 |
| G_tem_midin_r | 1.0174 | 0.9945 | 0.9945 | 0.2882 | 0.2288 | 0.2563 |
| G_tem_midin_l | 1.0331 | 0.9999 | 0.9959 | 0.2576 | 0.2270 | 0.1936 |
| G_fus_r | 0.8763 | 0.9028 | 0.9426 | 0.2011 | 0.3760 | 0.3116 |
| G_fus_l | 0.8737 | 0.9072 | 0.9241 | 0.2705 | 0.4300 | 0.3009 |
| Post_TL_l | 1.0509 | 0.9653 | 0.9514 | 0.2031 | 0.2017 | 0.1927 |
| Post_TL_r | 1.0686 | 1.0031 | 0.9806 | 0.1810 | 0.3407 | 0.2392 |
| G_sup_temp_ant_l | 0.8336 | 0.8197 | 0.8596 | 0.3017 | 0.2578 | 0.2951 |
| G_sup_temp_ant_r | 0.8331 | 0.8533 | 0.8872 | 0.2571 | 0.2431 | 0.3103 |
| PL_postce_G_l | 1.0402 | 0.9952 | 0.9967 | 0.2619 | 0.2021 | 0.2058 |
| PL_postce_G_r | 1.0960 | 1.0962 | 1.0974 | 0.2743 | 0.2649 | 0.1721 |
| PL_sup_pa_G_l | 1.1702 | 1.0494 | 1.0205 | 0.3483 | 0.3650 | 0.3759 |
| PL_sup_pa_G_r | 1.1713 | 1.0603 | 1.0338 | 0.3407 | 0.3902 | 0.3591 |
| PL_rest_l | 1.0818 | 0.9018 | 0.8833 | 0.3089 | 0.3227 | 0.3423 |
| PL_rest_r | 1.1134 | 0.9834 | 0.9552 | 0.2728 | 0.4205 | 0.3472 |
| OL_rest_lat_l | 1.1491 | 1.0147 | 0.9638 | 0.2089 | 0.4025 | 0.3991 |
| OL_rest_lat_r | 1.1715 | 1.0612 | 1.0025 | 0.2046 | 0.5190 | 0.3976 |
| OL_ling_G_l | 1.2047 | 1.2020 | 1.1714 | 0.2230 | 0.2014 | 0.2159 |
| OL_ling_G_r | 1.1960 | 1.2253 | 1.2028 | 0.2581 | 0.2606 | 0.2496 |
| OL_cuneus_l | 1.2921 | 1.1993 | 1.1494 | 0.2145 | 0.2790 | 0.3139 |
| OL_cuneus_r | 1.3141 | 1.2231 | 1.1776 | 0.2407 | 0.3965 | 0.3445 |
| Insula_l | 1.0199 | 1.0600 | 1.1104 | 0.2216 | 0.4783 | 0.3497 |
| Insula_r | 1.0386 | 1.1087 | 1.1452 | 0.3003 | 0.4577 | 0.3650 |
| CaudateNucl_l | 0.8619 | 0.7228 | 0.7817 | 0.2680 | 0.4396 | 0.1930 |
| CaudateNucl_r | 0.6729 | 0.4833 | 0.5392 | 0.2587 | 0.3917 | 0.1852 |
| NuclAccumb_l | 1.0022 | 0.9962 | 1.0453 | 0.2366 | 0.4823 | 0.3109 |
| NuclAccumb_r | 0.8437 | 0.7711 | 0.8303 | 0.2513 | 0.4758 | 0.3370 |
| Putamen_l | 1.2010 | 1.2355 | 1.2735 | 0.2772 | 0.4412 | 0.2914 |
| Putamen_r | 1.1647 | 1.1371 | 1.1711 | 0.2667 | 0.5072 | 0.3050 |
| Thalamus_l | 0.9862 | 0.9069 | 0.9597 | 0.2653 | 0.5377 | 0.2233 |
| Thalamus_r | 0.9556 | 0.7996 | 0.8770 | 0.2548 | 0.4266 | 0.2222 |
| Pallidum_l | 0.9947 | 1.0632 | 1.1019 | 0.2770 | 0.3964 | 0.2343 |
| Pallidum_r | 0.8786 | 0.9369 | 0.9787 | 0.3570 | 0.4096 | 0.2647 |
| S_nigra_l | 0.9039 | 1.0074 | 1.0674 | 0.3190 | 0.3907 | 0.2496 |
| S_nigra_r | 0.9164 | 0.9747 | 1.0569 | 0.2430 | 0.4152 | 0.2405 |
| G_cing_ant_l | 0.9394 | 0.9730 | 1.0237 | 0.3382 | 0.2317 | 0.2220 |
| G_cing_ant_r | 0.9539 | 0.9713 | 1.0325 | 0.3232 | 0.2745 | 0.2193 |
| G_cing_post_l | 1.1255 | 1.1172 | 1.1440 | 0.3527 | 0.4468 | 0.3085 |
| G_cing_post_r | 1.1621 | 1.1872 | 1.2183 | 0.3412 | 0.4399 | 0.3276 |
| Hippocampus_r | 0.7773 | 0.8201 | 0.8672 | 0.3635 | 0.2352 | 0.2665 |
| Hippocampus_l | 0.7862 | 0.8522 | 0.8758 | 0.2956 | 0.3860 | 0.1955 |
| Amygdala_r | 0.7450 | 0.7862 | 0.8427 | 0.3194 | 0.2926 | 0.1785 |
| Amygdala_l | 0.7859 | 0.8855 | 0.9112 | 0.2575 | 0.4703 | 0.3396 |
| G_paraH_amb_r | 0.7696 | 0.7741 | 0.8238 | 0.3027 | 0.4711 | 0.3363 |
| G_paraH_amb_l | 0.8216 | 0.8996 | 0.9296 | 0.3030 | 0.3963 | 0.3265 |
| Subgen_antCing_l | 0.8228 | 0.8294 | 0.8698 | 0.3156 | 0.4956 | 0.3683 |
| Subgen_antCing_r | 0.8191 | 0.7512 | 0.8076 | 0.2466 | 0.4189 | 0.3262 |
| Subcall_area_l | 0.8391 | 0.8789 | 0.9448 | 0.3092 | 0.4710 | 0.3732 |
| Subcall_area_r | 0.7178 | 0.6891 | 0.7493 | 0.2718 | 0.4441 | 0.3516 |
| Presubgen_antCing_l | 0.9276 | 0.8973 | 0.9415 | 0.1679 | 0.4068 | 0.3298 |
| Presubgen_antCing_r | 0.9520 | 0.9177 | 0.9812 | 0.3044 | 0.4650 | 0.3137 |
| Cerebellum_l | 1.0309 | 1.1008 | 1.0887 | 0.2793 | 0.3922 | 0.3056 |
| Cerebellum_r | 1.0394 | 1.1210 | 1.0947 | 0.2795 | 0.2632 | 0.3075 |
| Brainstem | 0.8138 | 0.8989 | 0.9328 | 0.1709 | 0.2772 | 0.3096 |

**Supplemental Table 4** Average global mean scaled FDG-PET SUVr values (columns 2-4) and metabolic connectivity values (columns 5-7) of the three study cohorts (HC, DLB-DaT-, DLB-DaT+) for all 77 hammers regions (column 1).

**Supplemental Table 5** **–** PCA expression score comparison for different sites and scanners

| **A  HC** |  | Genova  (n=14) | Munich GE  (n=3) | Munich Biograph (n=6) |
| --- | --- | --- | --- | --- |
| Genova  (n=14) | p |  | >0.999 | 0.983 |
|  | η^2^ |  | 0.034 | 0.012 |
| Munich GE  (n=3) | p | >0.999 |  | 0.777 |
|  | η ^2^ | 0.034 |  | 0.012 |
| Munich  Biograph (n=6) | p | 0.983 | 0.777 |  |
|  | η ^2^ | 0.011 | 0.012 |  |

| **B DLB total** |  | Genova  (n=15) | Ljubljana  (n=7) | Leuven  (n=9) | Stockholm  (n=4) | Munich  Biograph  (n=32) | Munich mCT  (n=12) |
| --- | --- | --- | --- | --- | --- | --- | --- |
| Genova  (n=15) | p |  | 0.379 | 0.663 | 0.816 | 0.510 | 0.901 |
|  | η ^2^ |  | 0.129 | 0.027 | 0.012 | 0.031 | 0.002 |
| Ljubljana  (n=7) | p | 0.379 |  | 0.705 | 0.372 | 0.615 | 0.368 |
|  | η ^2^ | 0.129 |  | 0.253 | 0.242 | 0.022 | 0.209 |
| Leuven  (n=9) | p | 0.663 | 0.705 |  | 0.914 | 0.350 | 0.720 |
|  | η ^2^ | 0.027 | 0.253 |  | 0.001 | 0.082 | 0.021 |
| Stockholm  (n=4) | p | 0.816 | 0.372 | 0.914 |  | 0.454 | 0.831 |
|  | η ^2^ | 0.012 | 0.242 | 0.001 |  | 0.040 | 0.010 |
| Munich  Biograph (n=32) | p | 0.510 | 0.615 | 0.350 | 0.454 |  | 0.435 |
|  | η ^2^ | 0.031 | 0.022 | 0.082 | 0.040 |  | 0.044 |
| Munich mCT  (n=12) | p | 0.901 | 0.368 | 0.720 | 0.831 | 0.435 |  |
|  | η ^2^ | 0.002 | 0.209 | 0.021 | 0.009 | 0.044 |  |

| **C  DLB-DaT(-)** |  | Genova  (n=7) | Ljubljana  (n=1) | Leuven  (n=2) | Stockholm  (n=1) | Munich  Biograph  (n=6) | Munich mCT  (n=3) |
| --- | --- | --- | --- | --- | --- | --- | --- |
| Genova  (n=7) | p |  | NA | NA | NA | 0.920 | 0.772 |
|  | η ^2^ |  | NA | NA | NA | 0.024 | 0.011 |
| Ljubljana  (n=1) | p | NA |  | NA | NA  NA | NA | NA |
|  | η ^2^ | NA |  | NA |  | NA | NA |
| Leuven  (n=2) | p | NA | NA |  | NA | NA | NA |
|  | η ^2^ | NA | NA |  | NA | NA | NA |
| Stockholm  (n=1) | p | NA | NA  NA | NA |  | NA | NA |
|  | η ^2^ | NA |  | NA |  | NA | NA |
| Munich Biograph (n=6) | p | 0.920 | NA | NA | NA |  | >0.999 |
|  | η ^2^ | 0.024 | NA | NA | NA |  | 0.052 |
| Munich mCT  (n=3) | p | 0.772 | NA | NA | NA | >0.999 |  |
|  | η ^2^ | 0.011 | NA | NA | NA | 0.052 |  |

| **D  DLB-DaT(+)** |  | Genova  (n=8) | Geneva  (n=14) | Ljubljana  (n=6) | Leuven  (n=7) | Stockholm  (n=3) | Lingkoping  (n=10) | Munich  GE  (n=3) | Munich  Biograph  (n=26) | Munich  mCT  (n=9) |
| --- | --- | --- | --- | --- | --- | --- | --- | --- | --- | --- |
| Genova  (n=8) | p |  | 0.768 | >0.999 | 0.831 | 0.900 | 0.953 | 0.799 | 0.947 | 0.957 |
|  | η ^2^ |  | 0.064 | 0.136 | 0.075 | 0.072 | 0.001 | 0.104 | 0.008 | 0.020 |
| Geneva  (n=14) | p | 0.768 |  | >0.999 | 0.966 | >0.999 | 0.815 | 0.924 | 0.990 | 0.980 |
|  | η ^2^ | 0.064 |  | 0.227 | 0.000 | 0.007 | 0.038 | 0.004 | 0.094 | 0.004 |
| Ljubljana  (n=6) | p | >0.999 | >0.999 |  | >0.999 | 0.831 | 0.956 | >0.999 | 0.798 | >0.999 |
|  | η ^2^ | 0.136 | 0.227 |  | 0.239 | 0.181 | 0.037 | 0.238 | 0.038 | 0.141 |
| Leuven  (n=7) | p | 0.831 | 0.966 | >0.999 |  | 0.952 | 0.923 | 0.953 | >0.999 | 0.984 |
|  | η ^2^ | 0.075 | 0.000 | 0.239 |  | 0.007 | 0.033 | 0.003 | 0.071 | 0.005 |
| Stockholm  (n=3) | p | 0.900 | >0.999 | 0.831 | 0.952 |  | 0.986 | 0.972 | 0.941 | >0.999 |
|  | η ^2^ | 0.072 | 0.007 | 0.181 | 0.007 |  | 0.034 | 0.001 | 0.058 | 0.014 |
| Lingkoping  (n=10) | p | 0.953 | 0.815 | 0.956 | 0.923 | 0.986 |  | 0.975 | 0.982 | 0.920 |
|  | η ^2^ | 0.001 | 0.038 | 0.037 | 0.033 | 0.034 |  | 0.030 | 0.002 | 0.015 |
| Munich GE  (n=3) | p | 0.799 | 0.924 | >0.999 | 0.953 | 0.972 | 0.975 |  | 0.868 | >0.999 |
|  | η ^2^ | 0.104 | 0.004 | 0.238 | 0.003 | 0.001 | 0.030 |  | 0.056 | 0.012 |
| Munich Bio  (n=26) | p | 0.947 | 0.990 | 0.798 | >0.999 | 0.941 | 0.982 | 0.868 |  | 0.900 |
|  | η ^2^ | 0.008 | 0.094 | 0.038 | 0.071 | 0.058 | 0.002 | 0.056 |  | 0.040 |
| Munich mCT  (n=9) | p | 0.957 | 0.980 | >0.999 | 0.984 | >0.999 | 0.920 | >0.999 | 0.900 |  |
|  | η ^2^ | 0.020 | 0.004 | 0.141 | 0.005 | 0.014 | 0.015 | 0.012 | 0.040 |  |

**Supplemental Table 5** Z-scores of PCA expression scores were compared between differing imaging sites and differing scanner types to test for a significant influence on the obtained results. Results of t-tests including false discovery rate correction (p-value) and effect sizes (η ^2^) are presented in four separate crosstabs for the respective cohorts (HC, total DLB cohort consisting of DLB-DaT(+) and DLB-DaT(-), as well as both of the DLB subgroups, DLB-DaT(+) and DLB-DaT(-)). T-tests were performed for all conditions with n>2 scans.
