# Supplementary material for: Metabolic network alterations as a supportive biomarker in dementia with Lewy bodies with preserved dopamine transmission
Source: Eur J Nucl Med Mol Imaging. 2023 Nov 16;51(4):1023–34. doi: 10.1007/s00259-023-06493-w (PMC10881642; doi:10.1007/s00259-023-06493-w)
Supplement: Supplementary file 2 — Supplementary file2 (DOCX 47 KB) [file 259_2023_6493_MOESM2_ESM.docx]

**Step by Step guide for PCA expression score calculation.**

1. A principal component analysis (PCA) needs to be conducted using the FDG-PET values (Hammers, logarithmic and centered) of the training set (23 HC + 86 DLB-DaT(+)) provided in sheet 2 of the included Excel file and corresponding FDG-PET values of the single subject in question. SPSS or other software solutions need to be used to conduct the PCA.

2. The 12 resulting PCs from this analysis need to be extracted and copied into a free column in sheet 1 of the provided Excel file (see demonstrated below in yellow for a DLB-DaT(-) subject in column C).


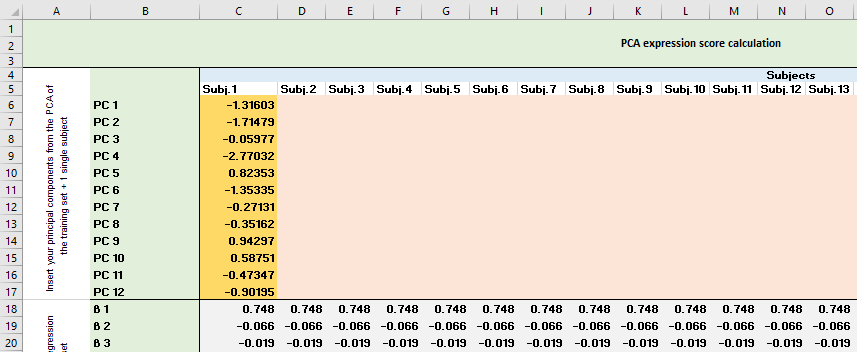


3. The Calculation Sheet will automatically weight the PCs from the PCA with the regression factors β1-12 that were extracted from PCA regression analysis of the provided training set (rows 18-29).

4. The sum of the weighted PCs (automatically calculated in lines 43/44, see below in red) is the expression score for the subject in question and can be interpreted by comparison with expression scores from HCs and other neurodegenerative diagnoses.


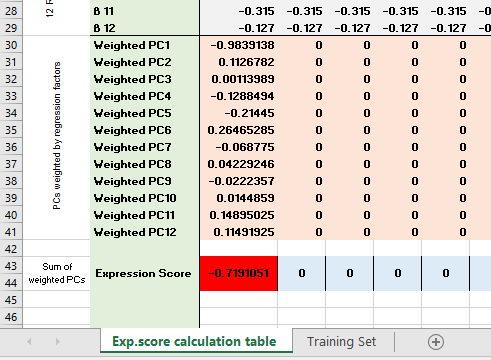


5. To provide more stability and prevent floating scores, transformation of PCA expression scores into z-scores is recommended, using HC expression scores as comparative basis:

*Z-score = (expression score of single subject - mean expression score of HCs) / standard deviation of expression scores of HCs*
